# School-based interventions to promote adolescent health: A systematic review in low- and middle-income countries of WHO Western Pacific Region

**DOI:** 10.1371/journal.pone.0230046

**Published:** 2020-03-05

**Authors:** Tao Xu, Sachi Tomokawa, Ernesto R. Gregorio, Priya Mannava, Mari Nagai, Howard Sobel

**Affiliations:** 1 National Center for Women and Children's Health, Chinese Center for Disease Control and Prevention, Beijing, China; 2 Reproductive, Maternal, Newborn, Child and Adolescent Health, Division of Noncommunicable Diseases and Health Through Life-Course, World Health Organization, Regional Office for the Western Pacific, Manila, Philippines; 3 Faculty of Education, Shinshu University, Nagano, Japan; 4 Health Promotion Unit, Prevention of Noncommunicable Diseases, Noncommunicable Diseases and Mental Health, World Health Organization, Geneva, Switzerland; 5 Department of Health Promotion and Education, College of Public Health, University of the Philippines, Manila, Philippines; 6 Department of Global Health, Graduate School of Health Sciences, University of the Ryukyus, Nishihara, Japan; 7 Bureau of International Cooperation, National Center for Global Health and Medicine, Tokyo, Japan; Fordham University, UNITED STATES

## Abstract

**Background:**

In the World Health Organization Western Pacific Region (WHO WPRO), most adolescents enroll in secondary school. Safe, healthy and nurturing school environments are critical for adolescent health and development. Yet, there were no systematic reviews found on the efficacy of school-based interventions among adolescents living in low and middle income countries (LMIC) in the Region. There is an urgent need to identify effective school-based interventions and facilitating factors for successful implementation in adolescent health in WPRO.

**Methods:**

For this systematic review, we used five electronic databases to search for school-based interventions to promote adolescent health published from January 1995 to March 2019. We searched RCT and non-RCT studies among adolescents between 10 to 19 years old, done in LMIC of WHO WPRO, and targeted health and behaviour, school environment and academic outcomes. Quality of studies, risk of bias and treatment effects were analyzed. Effective interventions and implementation approaches were summarized for consideration in scale-up.

**Results:**

Despite a broad key term search strategy, we identified only eight publications (with 18,774 participants). Most of the studies used knowledge, attitudes and behaviours as outcome measures. A few also included changes in the school policy and physical environment as outcome measures while only one used BMI, waist circumference and quality of life as their outcome measures. The topics in these studies included: AIDS, sexual and reproductive health, de-worming, nutrition, obesity, tobacco use, and suicide. Some interventions were reported to be successful in improving knowledge, attitudes and behaviours, but their impact and scale were limited. The interventions used by the different studies varied from those that addressed a single action area (e.g. developing personal skills) or a combination of action areas in health promotion, e.g. developing a health policy, creating a supportive environment and developing personal skills. No intervention study was found on other important issues such as screening, counseling and developing safe and nurturing school environments.

**Conclusions:**

Only eight school-based health interventions were conducted in the Region. This study found that school-based interventions were effective in changing knowledge, attitudes, behaviors, healthy policies and environment. Moreover, it was clarified that policy support, involving multiple stakeholders, incorporating existing curriculum, student participation as crucial factors for successful implementation.

## Introduction

Numbering nearly 1.8 billion, more adolescents populated the earth than at any previous time in history [1]. In the World Health Organization (WHO) Western Pacific Region, one in five people (235 million) are adolescents [2]. While maternal and under-five mortality had decreased by around half, adolescent mortality has remained stagnant. Globally, an estimated 1.3 million adolescents died in 2012 from preventable causes such as road injuries, HIV/AIDS, suicide, lower respiratory infections and interpersonal violence [2]. Thus, as countries focused on limiting preventable deaths among women and children for the Millennium Development Goals, Sustainable Development Goal 3.15 requires us to also focus on adolescents [2]. Adolescence is a period of experimentation and maturation. It is a time of physical, psychological, and social transitions from childhood to adulthood. Many unhealthy habits driving the non-communicable disease epidemic begin in adolescence. Thus, establishing healthy habits in adolescence is critical.

Today’s generation of adolescents faces a different world from what their parents and grandparents had. New political, economic, educational, technological and religious realities shape the world in which adolescents today live. They change the way adolescents transition from childhood to adulthood [3]. Understanding how to best support adolescents to have a healthy and smooth transition, we need to understand both how the transitions occur and how the external forces interact with the transitions.

The Lancet Commission has recommended the development and practice of effective evidence-based policies and interventions to reduce the burden of adolescent mortality and morbidity worldwide [4]. As adolescents spend a large proportion of their time at schools, they should be an important place to support adolescent health and development. In 2013, the net enrollment rate of primary and secondary school in the Western Pacific Region was 94.3% and 78.5% respectively [5]. Thus, schools have the potential to reach a vast majority of adolescents and school health education has the potential to positively impact a large proportion of youth who rarely visit health facilities. In low-and middle-income countries, school health is additionally cost-effective, and improves the effectiveness of their general education [6]. For these reasons, school-based health interventions become a major area of focus for the WHO.

Since its launch in 1995, the WHO's Global School Health Initiative has sought to mobilize and strengthen school health programs globally. In effect, school health programs should strive to formulate health policies and provide safe and healthy environments, health education, and health services including screening for various conditions and behaviours. However, existing evidence on the impact of school health programs on adolescent health and development is limited. Currently, countries use the global school-based student health survey (GSHS), a national level surveillance project designed to help them measure and assess the behavioural risk and protective factors in 10 key areas among young people aged 13 to 17 years. However, data on school health service implementation and utilization is not well collected. A WHO Cochrane review, which synthesized 67 cluster trials on school health-promoting interventions, found little or no evidence of effectiveness in reducing obesity, fat intake, alcohol use, drug use, mental health problems, violence or bullying. Furthermore, 88% of the identified trials came from high-income countries [7]. Another review presented a global overview of school health service using data from 102 countries. However, 71.6% of interventions came from high-and upper-middle-income countries, and no interventions were reported addressing important causes of mortality and ill health in adolescents as listed above [8]. A scoping review of 30 school-based health interventions in developing countries found significant increase in knowledge, beliefs and intentions, but no improvement in health behaviors and outcome [9]. Shackleton et al conducted a systemic review of 22 reviews on school-based health interventions and found 77% of them were conducted in the United States. The authors found little evidence that interventions such as sexual-health clinics, anti-smoking policies and other approaches targeting at-risk students were effective [10]. None of the above mentioned literature focuses only on adolescents.

In 2015, The United Nations (UN) extended the existing Every Women, Every Child agenda to include adolescents through the Global Strategy for Women’s, Children’s and Adolescents’ Health. This strategy called for evidence-based interventions to address the health and developmental needs of adolescents. Since nearly 90% of adolescents live in low- and middle-income countries (LMICs)[1], it is therefor critical to invest more in health to meet their needs. A recently published Lancet article highlighted the need to focus on screening, counseling and treating adolescents for common morbidities and risk behaviors that had long-term impact on well-being [11]. Member states in the WHO Western Pacific Region have raised demands for evidence-based interventions that can guide national actions. However, we found no systematic reviews of school-based interventions for adolescents in the Region. We conducted this review to describe the characteristics and identify effectiveness of school-based intervention and facilitating factors for successful intervention to promote adolescent health in low and middle income countries of WHO Western Pacific Region.

## Methods

### Criteria for considering studies for this review

#### Types of interventions

Health related interventions in primary or secondary schools were included, such as health promotion, screening for health and psychosocial conditions, creating safe and nurturing learning environments. Studies addressing any of these interventions were included. In some instances, interventions were implemented in community and home settings as well as the school, but only the component for schools was included.

#### Types of studies

Due to the limited availability of randomized controlled trials (RCTs), both RCTs and non-RCTs were included in this review. Non-RCTs had at least pre-and post-test to evaluate the effects of the intervention.

#### Types of participants

Studies that included school-attending adolescents between the ages of 10 to 19 years were included. Some studies included young children in the intervention (e.g. 6 to 18 years) but we included only those over 10 in the analysis where the data was available.

#### Countries of focus

Studies conducted in countries and areas in the Western Pacific Region were included in this review. The primary focus was low-and middle-income countries, as defined by the World Bank. These countries included Cambodia, China, Fiji, Kiribati, Lao PDR, Malaysia, Marshall Islands, Federated States of Micronesia, Mongolia, Palau, Papua New Guinea, the Philippines, Samoa, Solomon Islands, Tonga, Tuvalu, Vanuatu, and Viet Nam. The second search included all LMICs globally within the education database (ERIC).

#### Types of outcome measures

The range of topics included in the review included two categories, based on the health promotion framework whereby health is promoted throughout the whole school environment [7]: 1) Health and behaviour outcomes; and 2) School environment and academic outcomes. Studies were excluded if they did not present any outcome measures as mentioned below. Specifically, interventions that only focused on health-related knowledge and attitude in the absence of impact on health, behaviour, school environment or academic achievement were excluded.

1) Health and behaviour outcomes included:

Obesity, overweight or body size per body mass index, height-for-age, weight-for-age, and weight-for-height z-scores; self-reported levels of physical activity or sedentary behaviours; self-reported food intake (consumption of fruits and vegetables, high fat or sugar foods), indicators of specific nutritional deficiencies (iron, iodine, and vitamin A deficiencies), disordered eating habits; incidence of diarrhoea, cold or influenza, pneumonia, skin disease, worms, head lice; incidence of traffic accidents or other accidents or injuries in school or at home; oral health (e.g., decayed, missing or filled teeth index), self-reported dental hygiene behaviours such as regular tooth brushing; self-reported use of cigarettes or other tobacco products, alcohol or other drugs (legal or illegal); incidence of sexually transmitted infections, pregnancy or abortion, self-reported use of condoms or other contraception, abstinence or delaying of sexual intercourse; well-being or quality of life, incidence of self-harm or suicide, self-reliance, coping skills, learning engagement self-esteem, depression, family connectedness, peer support; self-reported violence (for example, got into a fight); self-reported incidence of being bullied or bullying others.

2) School environment and academic outcomes included:

Academic scores, dropout rate, attendance rate; change of number of water and sanitation facilities, health-related school policies; ratings of school climate, attachment to school, satisfaction with school, change of school curriculum.

An example of the detailed search strategies is shown in Supporting Information (S1 Table).

### Search strategy

#### Electronic search

The search strategy followed the Cochrane Collaboration methodology for conducting a comprehensive search of the literature [12]. We used a combination of four categories of searching terms, including school base (i.e. school, student, classroom, college), adolescent (i.e. adolescent, child, young people), name of WPRO LMI Countries, and intervention study (i.e. intervention, trial, program). The detailed search strategy is listed in Appendix 1. The first search was conducted in September 2015 and the second search in March 2019. Publications from January 1995 to March 2019 were included. Studies were not excluded on the basis of language. Four electronic health and medicine databases were initially searched: Cochrane Central Register of Controlled Trials (CENTRAL), PubMed, Social Science Citation Index (Web of Knowledge), and Western Pacific Region Index Medicus (WPRIM). The Education Resources Information Centre (ERIC) database was searched in both rounds of search for all studies in LMICs.

#### Searching other resources

Additional literature was identified by searching the Google Scholar, the excluded meta-analysis or review papers, and the reference lists of included studies. Experts in the field of adolescent health and school-based intervention were also contacted with a view to seeking additional references.

### Data collection and analysis

#### Selection of studies

All citations were downloaded into Online Endnote. Duplicate titles were eliminated. Two reviewers screened titles, abstracts, and lastly full texts based on the criteria for considering studies for this review. Disagreements were resolved through discussion between the two reviewers. In the advent disagreement remained, a third person was available to arbitrate.

#### Data extraction and management

Two reviewers independently completed standardized data extraction forms. Information extracted included: 1) First author, country or area, and year of publication; 2) Setting: public versus private school, rural versus urban, level of school (primary, secondary), size of school; 3) Participants: sample size, age, gender (for both intervention and control populations, where applicable); 4) Study design (RCT, non-RCT); 5) Intervention characteristics (health focus, duration, content and activities, both intervention and control population, if applicable); 6) Health topics of concern; 7) Outcome measures (subjective or objective measures, short term or long term, effect size, if applicable); 8) Lessons learnt.

#### Assessment of risk of bias in included studies

We assessed risk of bias within each study using the tool adapted from the Cochrane Handbook for Systematic Reviews of Interventions. Two reviewers independently judged the likelihood of bias in the following five domains.

1) Selection bias: systematic differences between baseline characteristics of the groups that are compared; 2) Performance bias: systematic differences between groups in the care that is provided, or in exposure to factors other than the interventions of interest; 3) Detection bias: systematic differences between groups in how outcomes are determined; 4) Attrition bias: systematic differences between groups in withdrawals from a study. Withdrawals from the study lead to incomplete outcome data. Attrition refers to situations in which outcome data are not available; 5) Reporting bias: Systematic differences between reported and unreported findings.

#### Measure of treatment effect

No calculations were performed. All data was presented in the format mean and standard deviation (SD) with 95% confidence intervals (CI), if provided. Results with P>0.05 were reported as not significant.

Data synthesis

Each study was summarized and described according to variables such as type and activities of intervention, targeted population, follow-up and outcome effects. Common components of interventions were summarized and grouped. Lessons learnt from each study in terms of intervention implementation were identified. Effective interventions and implementation approaches for different health issues were summarized for consideration in scale-up.

### Patient and public involvement

This research was done without patient and public involvement.

## Results

### Selection of studies

**Fig 1:**
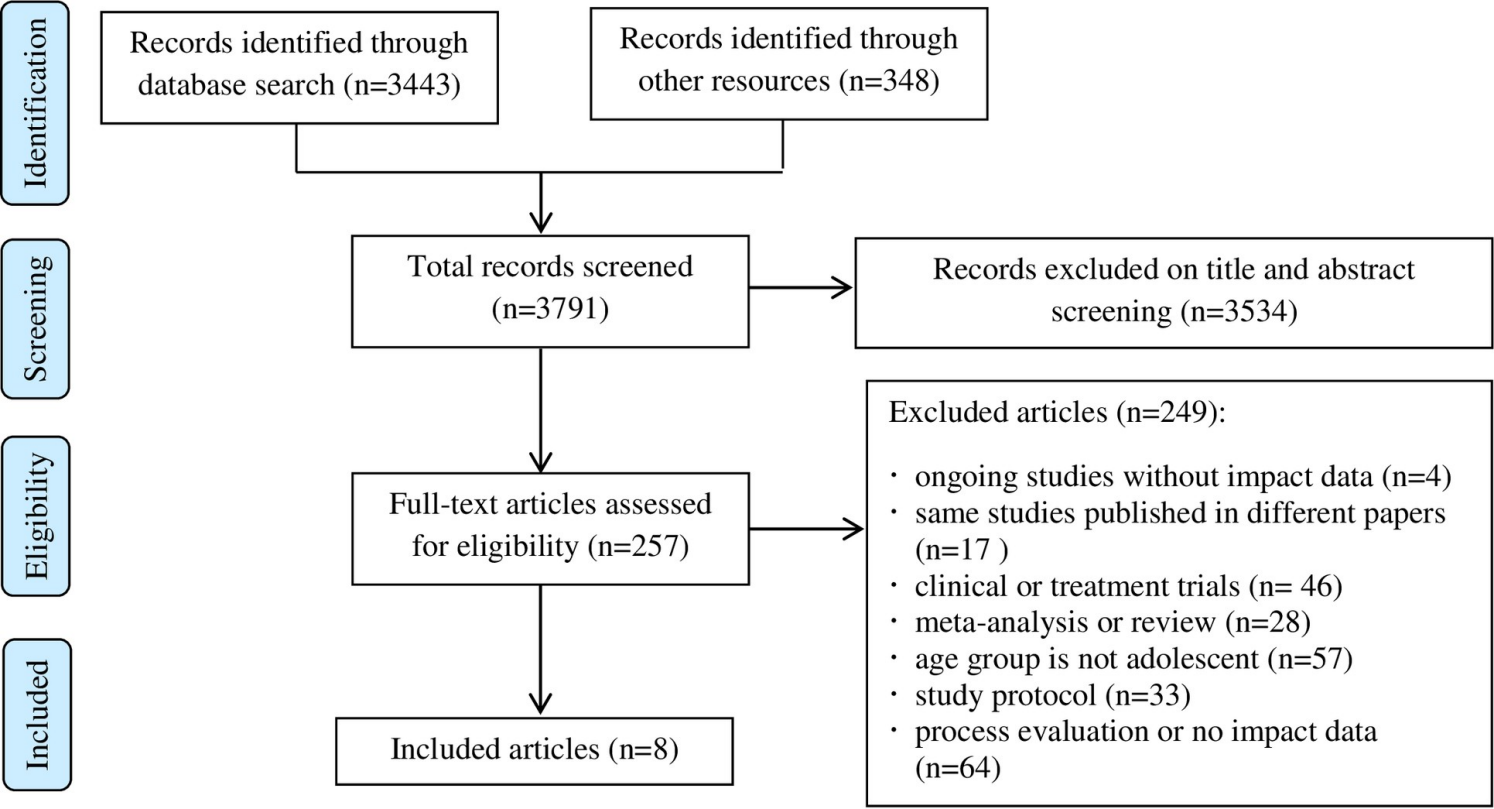
Search and selection process of the articles.

The initial database search identified 3791 articles of which 3534 were excluded based on title and abstract screening. Expanding the search using the ERIC database to all LMICs globally yielded no additional articles that met the inclusion criteria. This left 257 full-text articles that were assessed for eligibility. Eight articles met the eligibility criteria for inclusion in the review (Fig 1). Although we tried to focus on school literature, all eight articles were published in health-related journals (two in Health Promotion International, one in AIDS, one in the Journal of Adolescent Health, one in Addictive Behaviors, one in International Journal of Epidemiology, one in Journal of Psychiatry and one in Malaysia Journal of Medical Science).

### Study overview

#### Study characteristics

Studies included in the review took place in China [13–16], the Philippines [17], Mongolia [18], Cambodia [19], and Malaysia [20]. Of the eight studies, three [13, 17,20] were cluster randomized controlled trials (CRCT), with schools in the study districts being randomly selected either for the intervention or the control group. The other five were non-randomized controlled trial (NRCT). In these studies, a convenient sampling method was used to select schools, based on the willingness of schools to participate, or the availability of school resources to implement the programme. Health topics of interventions included HIV/AIDS and sexual behaviors [17, 18], de-worming [15], nutrition [14], tobacco use [13,16], suicide [19], and obesity [20]. One study was funded by WHO [15], four by research foundations [13, 16, 17, 19], one by university grants [18]. Two study reported no funding sources [15,20]. The detailed information of the included studies is shown in Supporting Information (S2 Table).

#### Participants' characteristics

The eight studies that were included had 18,774 total participants with a sample size ranging from 97 to 4277. Five studies [13, 14, 16, 17,20] reported the average age of participants. One study [18] only reported the age range (15–19 years old) of students. Data stratified by age group was not provided in one study [15] containing both primary (6–12years old) and secondary (12–15years old) school students. One study only reported the participants as high school and secondary school students [19]. All studies included both sexes but data on distribution by sex was not available in three studies [14, 15, 17]. Of the five studies which reported distribution by sex, four [13, 16, 19, 20] had slightly more male than female respondents. The nutrition study was conducted in urban schools, and the de-worming study in rural schools. The area of the other three studies was not reported. One study was conducted in both public and private schools [16] and the others were done in government or public schools.

#### Intervention characteristics

Five studies [13, 14, 15, 16, 18] focused on the long-term results of the intervention, with the intervention duration ranging from 12 to 36 months. One study [19] focused on medium-term intervention with the duration of six months and two focused on short-term interventions of 6 to 12 weeks [17,20]. Three studies [13, 14, 15] employed the Health Promoting School Framework in their interventions, which included health policy, health curriculum, health environment, and health skills training in the intervention package. One study used the PRECEDE-PROCEED framework in intervention design. The other studies only used peer-or teacher-led health skill training. Two studies focused on multiple risk behaviors such as sexual behaviors and alcohol or drug use [13], hand washing, eating and exercise behaviors [15]. Six studies only addressed single behaviors. None of the eight studies focused on screening for health conditions. All studies focused on health promotion interventions (e.g. behavior change, infectious diseases), with two of them [14, 15] also addressing safe learning environments (water and sanitation facilities).

### Components of interventions

#### Health education and training

All studies employed training and educational activities to change the knowledge, attitude and behaviors of students. Peer-education/peer-influence module was used in three studies [13, 16, 18]. The other studies provided training via teachers or professionals (psychologists, nurses), one of which used the web-based interactive tools for the training [20]. The authors noted that gender- [19] and culture-specific [13] interventions might lead to better results and that peer activities in small group and informal formats [18] were more effective than in big and formal classes. Two studies [15, 16] incorporated health education curriculum into routine teaching agenda. Two other studies [14, 17] noted that health education should be established as a regular element of the curriculum.

#### Health-related school polices

Four studies looked at development of health-related school policies or regulation [13–16]. These policies ensured the concepts of HPS be accepted by government officials and community members [14], helped to integrate interventions into regular school curricula and community programs [12,15,16] and promoted funding to change school psychosocial environment [14]. In one study, schools established a working group comprised of the principal, teachers, parents, and community leaders to oversee the implementation of school policy [14]. In two other studies, tobacco control committees headed by the school principal were set up and regulations against smoking were set within the school [13, 16].

#### Changing school environment

In the three studies that employed the Health Promoting School framework, the school environment was reported to have improved. Schools in two studies renovated their grounds, improved the latrines and enhanced water supply facilities [14, 15]. Schools in the third study [16] simply placed non-smoking signs in the schoolyard. Authors in these three studies all believed these environmental changes do not only make the schools safer and healthier, but also contributed to the sustainability of the interventions.

#### Close collaboration among health and other sectors

All studies mentioned the importance of cooperation and coordination among health and other sectors. For example, both the HIV/AIDS and STD studies noted the importance of involving community religious sectors in developing training materials [13,16]. Both the de-worming and nutrition studies worked with municipal administration sectors to improve the latrines and water supply in the schools [14,15]. Two studies [13, 15] noted this collaboration was a requirement for the success of school-based interventions. In two studies, the involvement of the community, especially parents, was also noted as an important facilitator of successful implementation [13, 17].

#### Scaling up to schools at the local and prefectural level

The authors of three studies [14, 15, 19] analyzed the possibility of scaling up the intervention. Scaling up of the deworming intervention was possible if the concept of HPS was acceptable to government officials [12]. School-based program working group comprised of the headmaster, teachers, parents and community leaders contributed to the scaling up of the nutrition intervention [13]. The Cambodia study focused on suicide prevention. Teachers who were engaged in the programme valued the process used in this intervention. The authors believed that if teachers were involved at early stage of programming and appreciate the process, then scaling up to national level may be possible [17]. In the China study on health promoting schools that focused on nutrition, the model was already expanded to 51 schools, representing about 93,000 students. Also, the curriculum content was expanded to cover all major adolescent health and development issues [20]. However, no impact evaluation of the change of health and academic performance was done.

#### Facilitating factors in implementing the interventions

A further extraction and synthesis of the intervention components revealed the facilitating factors of effective school health interventions. While health professionals play a key role in implementing health promoting programs, the involvement of communities, parents, teachers and principals is a facilitating factor for success. Supportive school environment and policies, including intervention contents into school curriculum are also preconditions for a successful intervention. When developing training materials, culture and gender factors should be considered. Students participation and skill-based education seem to be more effective approaches than health education classes or lectures given by teachers (Table 1).

**Table 1:** Facilitating factors in implementing the interventions.

Citation	Precondition			Stakeholder involvement			Approach			Content	
	Policy & environment	High quality training for teacher	Include in curriculum	Community&parents	Teacher, principal, educational authorities	Health professionals	Peer education	Students participate	Repeated intervention	Skill based education	Culture and gender attention
Aplasca et al., 1995		●	●	●	●	●			●	●	●
Xu et al., 2000	●			●	●	●		●		●	
Xia et al., 2004	●	●	●	●	●	●					
Cartagena et al., 2006			●			●	●	●		●	
Wen et al., 2010	●		●	●	●	●	●	●	●		
Chen et al., 2014	●					●	●	●	●		●
Jegannathan et al., 2014		●			●	●				●	●
Mohammed Nawi A et al., 2015						●		●			●

### Effects of interventions

All studies compared the effects at pre-and post-intervention or between the intervention and control groups. All studies reported significant knowledge and attitude changes. One study reported an 82.9% decrease in the multi-parasitism rate of helminth infection and 80.7% reduction of environment egg contamination [15]. One study reported a positive effect on handwashing behaviors (i.e. 'washing hand before eating' increased from 66.4% to 89.8% and 'washing hand after using toilet' increased from 87.5% to 93.6%) [14]. One study detected a significant behavioural change (condom use during sexual intercourse) but only in a sub-group of participants [18]. The Malaysia study reported no significant reduction on BMI, waist circumference, and body fat percentage after the 12 weeks obesity intervention [20].Three studies that employed the Health Promoting School framework reported qualitative data on changes in school policies and environment (Table 2).

**Table 2:** Summary of evidence.

Citation	Country	Study design*	Participants	Health topics	Intervention and control group	Outcome measures	Results
Aplasca et al., 1995	Philippines	CRCT	804 high school students (420 intervention, 384 control) Age: 13–16, average age is 14.7 in intervention and 14.9 in control.	AIDS	Intervention: Teacher-led AIDS training programme Control: No specific activities.	AIDS-related knowledge, attitudes; sexual behaviors and alcohol and drug use.	The difference of changes in mean scores of AIDS knowledge is +0.45 (AIDS biology), +0.93 (transmission) and +0.41 (prevention), with P<0.01. There was no statistically significant overall effect on intended preventive behavior.
Xu et al., 2000	China	NRCT	4063 intervention and 1050 control. Age: 6–12 for primary and 12–15 for secondary.	Deworming	Intervention: Examination and treatment of helminth infection, health education, improvement of physical environment, school policies and regulations Control: Examination and treatment of helminth infection	Knowledge & behaviors; prevalence of helminth infection; environmental egg contamination, School policy and environment.	Knowledge passing rate increased from 10.9% to 82.7% (P<0.005); behavior (–); multi-parasitism rate decreased from 42.8% to 7.3% (P<0.01); egg contamination rate declining by 80.7% (P<0.01);policy (+); Environment(+)
Xia et al., 2004	China	NRCT	4277 at baseline, 3346 at final evaluation. Average age intervention group 13.7, and 13.6 in control group.	Nutrition	Intervention: School-based working groups, nutrition training for school staff and students, student competitions, health promotion activities Control: No specific activities.	Knowledge, attitudes & behaviors, school policy, school environment.	Knowledge of "nutrient-rich foods" increased from 36.0% to 59.6%(P<0.01).Awareness: the importance of eating three adequate meals each day increased from 50.0% to 86.6% (P<0.01).Washing hands before eating increased from 66.4% to 89.8% (P<0.01), "washing hands after using toilet" increased from 87.5% to 93.6% (P<0.01);policy (+);environment(+)
Cartagenaet al., 2006	Mongolia	NRCT	320 interventions (M = 43%, F = 57%); 327 control (M = 43.5%, F = 56.5%). Secondary school, ages 15–19	HIV &SRH	Intervention: Peer education on HIV &SRH Control: No specific activities	Knowledge, attitudes, self-confidence & behaviors.	Small group peer education was more effective for knowledge (RI 5.03; 95% CI 3.08–8.21), attitude (RI 2.73; CI 95% 1.42–5.27), self-efficacy (RI 10.64,CI 8.59–13.19) and practice (OR 3.80, CI 95% 2.26–6.41)
Wen et al., 2010	China	NRCT	2343 7th and 8th grade students, control (1004) and intervention (1339). Boys accounts for 52.1%, girls 45.9%. Average age was 13.4.	Tobacco use	Intervention: PRECEDE-PROCEED model intervention using socio-ecological framework. Control: Basic once-a-year health curriculum.	Knowledge, attitudes & behaviours (ever or regular smoker)	Knowledge increased, with the effect size being 0.32 in 7th cohort (P<0.001) and 0.41 in 8th cohort (P<0.001). Reduced the probability of baseline experimental smokers’ escalating to regular smoker (7.9 vs 18.3%; adjusted odds ratio (OR) 0.34, 95% CI = 0.12–0.97), but did not reduce the probability of baseline non-smokers’ initiating smoking (7.9 vs10.6%; adjusted OR 0.86, 95% CI = 0.54–1.38). Did not reduce the probability of smoking initiation (P>0.05).
Chen et al., 2014	China	CRCT	709 Linzhi Tibetan (349 interventions, 360 controls) and 1098 Guangzhou Han (592 interventions, 506 controls). Average age was 14.5±1.1 years, 50.4% were boys.	Tobacco use	Intervention: Health policy in school; health environment in school and personal health skills. Control: No specific activities.	Knowledge, attitudes & behaviors, school policy, school environment.	Knowledge increased in Tibetan (β = 1.32, 95% CI (0.87–1.77) and Han groups (β = 0.47, 95% CI (0.11–0.83); attitudes toward smoking increased in Tibetan (β = 1.47, 95% CI (0.06–2.87)) but not in Han (β = −0.33, 95% CI (−1.68–1.01).Policy (+); environment (+).
Jegannathan et al., 2014	Cambodia	NRCT	168 interventions (M = 92, F = 76); 131 control (M = 53, F = 78). Secondary school, young people, data on age not available.	Suicide	Intervention: Life skill modules related to suicidal behaviors. Control: Three lessons on health, hygiene and nutrition	Attitude, life skills development scale.	Among high-risk boys, a small to moderate effect size on depressed (ES = 0.40), attention problems (ES = 0.46), aggressive behaviour (ES = 0.48) and externalizing syndrome (ES = 0.64). Cohen's D: 0.2 = small effect, 0.5 = moderate effect, 0.8 = large effect
Mohammed Nawi A et al., 2015	Malaysia	CRCT	47 intervention (M = 25, F = 22); 50 control (M = 30, F = 20). All participants were 12 years old.	Obesity	Intervention: Website based obesity information, interactive toolbar in the website for discussion and comments. Respondents were also advised to measure BMI every 2 weeks. Control: printed materials on the same information as in website.	BMI, waist circumference, Body fat, quality of life score	No significant reduction in BMI, waist circumference, and the body fat percentage between the intervention and control groups. The effect sizes of the reduction were too small (0.09, 0.11, and 0.09 for BMI, waist circumference and body fat percentage).

*CRCT = Cluster Randomized Controlled Trial, NRCT = Nonrandomized Controlled Trial.

### Risk of bias in the included studies

Selection bias included an assessment of both adequate sequence generation and allocation concealment. Five studies were assessed as having high risk of selection bias. Five studies were assessed as having unclear risk of detection bias because there was no blinding of participants. For the three studies that employed CRCT design, we assessed them as being at low risk of bias for random sequence generation and allocation concealment. The others were convenience samples of school and were assessed to have high risk of selection bias. Seven studies only evaluated self-reported indicators, so the risk of detection bias was unclear. Only one study reported laboratory examination results and was assessed as having low risk of detection bias [15] (Table 3).

**Table 3:** Risk of bias evaluation table*.

Citation	Selection bias		Performance bias	Detection bias	Attrition bias	Reporting bias
	Random sequence generation	Allocation concealment	Blinding of participants and personnel	Patient-reported outcomes	Long term (>6 wk)	Selecting reporting
Aplasca et al., 1995	–	–	–	?	+	–
Xu et al., 2000	+	+	?	–	–	+
Xia et al., 2004	+	+	?	?	–	–
Cartagena et al., 2006	+	+	?	?	–	–
Wen et al., 2010	+	+	–	?	–	–
Chen et al., 2014	–	–	–	?	–	–
Jegannathan et al., 2014	+	+	?	?	–	?
Mohammed Nawi A et al., 2015	–	–	?	?	–	–

^*^Categories for risk of bias are as follows: low risk of bias (–), unclear risk of bias (?), high risk of bias (+).

## Discussion

The overwhelming majority of adolescents attend school in LMICs in the WHO Western Pacific Region. Schools are a place where adolescents spend a large fraction of their lives. It should be a place that prepares and nurtures adolescents to transition from childhood to adulthood. Despite a vast and growing scientific literature on adolescent health and development, this systematic review found few studies on school-based interventions in LMICs in our region. Only eight studies met the inclusion criteria and all were published in health-related journals. This is despite a broad search strategy and expanding the search using the ERIC (educational) database to all LMICs globally.

The eight studies focused on AIDS, sexual and reproductive health, deworming, nutrition, tobacco use, obesity and suicide. No study focused on screening for health conditions or creating secure and nurturing learning environments. Only one study addressed life skills in promoting emotional well-being. However, the sample size of this study was small. While an acceptable large intervention effect size is above 0.8, the effect size for reducing aggressive behavior and externalizing syndrome among high-risk boys was only 0.48 and 0.64, respectively [19]. In addition, all studies were pilot research in nature. Although authors of three studies were optimistic that their interventions could be effectively scaled up to provincial or national level, however, no impact of the expansion was reported. It is unclear whether an impact evaluation was done or not and/or was not published because of negative results.

Adolescents face unique health and development issues [21, 22]. Evidence-based school health programmes should be aligned with their health priorities [23]. A previous study has shown that the important causes of mortality and health problems in adolescents such as mental health disorders, violence, injuries, and chronic conditions are neglected in school health services [8]. Therefore, school health programs should expand beyond traditional health education to support for the health and social transition of adolescents. These include screening for health and psychosocial conditions, providing counseling, creating safe and nurturing learning environments and treating for common morbidities and risk behaviors [24]. Screening adolescents to determine their well-being and to identify risky behaviours, and mental and physical health problems is a critical function for identifying barriers to a healthy transition to adulthood [2]. Screening needs to be linked with follow-up services, treatments and remediation of health problems [25]. Yet, we found no intervention studies on this critical topic.

Schools need to provide a safe, nurturing and healthy space for adolescents. However, schools in many countries in the region lack basic things like clean water, sufficient toilets and safe school grounds [26]. Schools should be a safe zone, a place of stability to counter the chaotic home environments where many adolescents come from. They should not be a place where teachers smoke in the classrooms, or where adolescent girls avoid school during the time of menstruation due to inadequate toilet facilities. They should be a place where students do not feel physically, sexually or psychologically threatened or neglected [27]. It should be a place where adolescents do not have physical hazards causing injury. It should be a place free of dangerous illnesses, where adolescents are immunized against common infectious diseases. Finally, it should be a place where adolescents develop resilience and achieve mental, spiritual and emotional well-being, and self-reliance through gaining life skills. We found some interventions addressed school physical environment, such as providing hygiene facilities. No study was found to address the nurturing school environments, which are creating psychosocial environment that enable adolescents’ learning and development and facilitate the emotional well-being.

Research from high income countries presented four key components of effective school health services: wide engagement with community, youth focus and participation, delivery of high-quality comprehensive care, and effective governance and administrative systems [28]. In our review, health education and health promotion are the most common interventions, and the Health Promoting School framework has been widely used in school-based programmes. Changing school health policies and environments, and incorporating health education into the curriculum are the key preconditions of success. School health policies should be developed to provide the schools with guidance on how to implement health promotion programs. Presence of health policies will not only contribute to program sustainability, but will likewise improve compliance and participation of teachers, students and other stakeholders to various adolescent school health programs[10].

Most of the studies noted the importance of involvement of stakeholders for a successful intervention. Involvement of school community members such as parents, religious leaders, teachers, principals may contribute to a sustainable mechanism for program implementation [10]. Changing school environment will also need the support and coordination from educational and/or municipal administrative sectors. Another facilitator of success is the use of peer education as an approach to deliver knowledge and promote behavior change among adolescents. Peer connections, peer modelling, and awareness of peer norms are protective against violence, substance use and risky sexual behaviors [22]. For example, Malaysian adolescents who had problems with peers were found to be more likely to suffer from depression [29]. However, training on positive peer relationships reduced bullying and depression among Korean adolescents [30]. These finding can provide valuable programmatic implications for counties in the Region to design effective interventions.

In our review, informal peer education with small groups was associated with not only increasing knowledge, but also slightly reducing risky sexual behaviours among secondary school students [18]. In the AIDS prevention study in the Philippines, the authors recognized that teaching about modern methods of contraception in the school might create a conflict between the Catholic Church and the Government. After discussion with school teachers, teachers' support for including condom use in the curriculum was obtained as an HIV preventive method rather than birth control method [17]. The study in Cambodia focused on suicide prevention. The same intervention showed improvement among high risk male adolescent but not among the girls. This indicates that gender-and culture-specific interventions might lead to better results [19].

## Limitations

The small number of studies included in the review hampers synthesizing data and drawing generalizable conclusions that apply to all low- and middle-income countries in the Region. While studies were not excluded on the basis of language, most of the available databases used are English-dominated. Given the great diversity of languages in our Region, some studies written in local languages might exist which we could not locate. All eight studies had a least one quality concern, be in small sample size, low effect size or high risk of research bias. Lastly, often a huge gap exists between practice and research. For example, since the late 1990s, international non-government organizations and government agencies had implemented at least 14 different school-based programmes in Cambodia. However, only four papers were published after the year 2000. In addition, many school-based health studies focused on evaluating the process rather than impact. In our review, around a quarter of full-text studies assessed for eligibility were excluded because of the absence of impact data. Thus, a cautious interpretation of intervention effectiveness is needed.

## Interpretations

Since the launch of the WHO report on Health for the World’s Adolescent in 2014, Member States in the WHO Western Pacific Region have raised demands for evidence-based actions to promote the health and development of adolescents. Schools are a place where adolescents spend a large fraction of their lives. It should be a place that prepares and nurtures them to transition from childhood to adulthood. Despite the critical importance of adolescents attending safe, nurturing and healthy schools, our review found only eight school-based health interventions conducted in LMICs in the Region. It is noteworthy that no health-related interventions were found in any LMICs globally in the education literature. Some countries have reported successful interventions for adolescent health, but the effect size is low, the impact is limited and the results have high risk of bias. Despite these limitations, current studies have contributed to provide evidence on facilitating factors in implementing effective interventions. These findings will enrich the limited body of scientific evidence and help each country to contextualize and scale up school based interventions. Future studies are needed to evaluate the long-term effectiveness of interventions addressing the health needs of adolescents, focusing on integrated strategies to improve early screening, diagnosis, treatment and referral and creating secure and nurturing learning environments.

## Supporting information

S1 Table Search strategy (Web of Science). (DOCX)

S2 Table Overview of study characteristics. (DOCX)

S1 File The PRISMA checklist. (DOC)

## References

[1] UNFPA. State of world population. 2014. Available at: http://www.unfpa.org/swop (accessed January 10, 2019)

[2] WHO. Health for the World’s Adolescents Report. 2014. Available at: http://www.who.int/maternal_child_adolescent/topics/adolescence/second-decade/en/ (accessed February 2, 2019).

[3] Fatusi AO, Hindin MJ. Adolescents and youth in developing countries: Health and development issues in context. *Journal of Adolescence* 2010; 33: 499–508. 10.1016/j.adolescence.2010.05.019 20598362

[4] Patton GC, Sawyer SM, Santelli JS, Ross DA, Afifi R, Allen NB, et al Our future: a Lancet commission on adolescent health and wellbeing. *Lancet* 2016 387: 2423–2478. 10.1016/S0140-6736(16)00579-1 27174304 PMC5832967

[5] UNESCO Institute for Statistics. Regional and country profiles. 2013.Available at: http://www.uis.unesco.org/Pages/default.aspx (accessed March 20, 2019)

[6] Jimba M, Poudel KC, Poudel-Tandukar K, Wakai S. School health research in low-income countries in East Asia and the Pacific. JAMJ 2005; 8 (4): 168–174.

[7] Langford R, Bonell CP, Jones HE, Pouliou T, Murphy SM, Waters E, et al,. The WHO health promotion school framework for improving the health and well-being of students and their academic achievement. Cochrane Database of Systematic Review 2014; Issue 4. Art. No.: CD008958. 10.1002/14651858.CD008958.pub2 24737131 PMC11214127

[8] Baltag V, Pachyna A, Hall J. Global Overview of School Health Services: Data from 102 Countries. Health Behavior and Policy Review 2015; 2 (4): 268–283.

[9] Mukamana O, Johri M. What is known about school-based interventions for health promotion and their impact in developing countries? A scoping review of the literature. Health Education Research 2016; 31(5): 587–602. 10.1093/her/cyw040 27516095

[10] Shackleton N, Jamal F, Viner RM, Dickson K, Patton G, Bonell C. School-based interventions going beyond health education to promote adolescent health: systematic review of reviews. Journal of Adolescent Health 2016; 58: 382–96. 10.1016/j.jadohealth.2015.12.017 27013271

[11] Azzopardi PS, Hearps SJC, Francis KL, Kennedy EC, Mokdad AH, Kassebaum NJ, et al Progress in adolescent health and wellbeing: tracking 12 headline indicators for 195 countries and territories, 1990–2016. *Lancet* 2019; 393:1101–1118. 10.1016/S0140-6736(18)32427-9 30876706 PMC6429986

[12] Higgins JPT, Green S, eds. Cochrane Handbook for Systematic Reviews of Interventions, version 5.1.0 [updated March 2011]. The Cochrane Collaboration. Available at: www.cochrane-handbook.org.

[13] Chen L, Chen Y, Hao Y, Gu J, Guo Y, Ling W. Effectiveness of school-based smoking intervention in middle school students of Linzhi Tibetan and Guangzhou Han ethnicity in China. *Addictive Behaviors* 2014; 39: 189–195. 10.1016/j.addbeh.2013.09.026 24129264

[14] Xia S, Zhang X, Xu S, Tang S, Yu S, Aldinger C, et al Creating health-promoting schools in China with a focus on nutrition. Health Promotion International 2004; 19 (4): 409–418. 10.1093/heapro/dah402 15520042

[15] Xu L, Pan B, Lin J, Chen L, Yu S, Jack J. Creating health-promoting schools in rural China: a project started from deworming. *Health Promotion International* 2000; 15 (3): 197–206.

[16] Wen X, Chen W, Gans KM, Golby SM, Lu C, Liang C, et al Two-year effects of a school-based prevention programme on adolescent cigarette smoking in Guangzhou, China: a cluster randomized trial. *International Journal of Epidemiology* 2011; 39(3): 860–876. 10.1093/ije/dyq001 PMC4817589 20236984

[17] Aplasca MR, Siegel D, Mandel JS, Santana-Arciaga RT, Paul J, Hudes ES, et al Results of a model AIDS prevention program for high school students in the Philippines. *AIDS* 1995; 9, S7–S13. 8562004

[18] Cartagena RG, Veugelers PJ, Kipp W, Magigav K, Laing LM. Effectiveness of an HIV prevention program for secondary school students in Mongolia. *Journal of Adolescent Health* 2006; 39: 925 e9–925.e16. 10.1016/j.jadohealth.2006.07.017 17116526

[19] Jegannathan B, Dahlblom K, Kullqren Gl. Outcome of a school-based intervention to promote life-skills among young people in Cambodia. *Asian Journal of Psychiatry* 2014; 9: 78–84. 10.1016/j.ajp.2014.01.011 24813042

[20] Mohammed Nawi A, Che Jamaludin FI. Effect of Internet-based Intervention on Obesity among Adolescents in Kuala Lumpur: A School-based Cluster Randomised Trial. Malays J Med Sci. 2015; 22(4): 47–56. 26715908 PMC4683849

[21] Sawyer SM, Afifi RA, Bearinger LH, Blakemore SJ, Dick B, Ezeh AC, et al Adolescence: a foundation for future health. *Lancet* 2012; 379: 1630–1640. 10.1016/S0140-6736(12)60072-5 22538178

[22] Viner RM, Ozer EM, Denny S, Marmot M, Resnick M, Fatusi A, et al Adolescence and the social determinants of health. *Lancet* 2012; 379: 1641–1652. 10.1016/S0140-6736(12)60149-4 22538179

[23] Salam RA, Das JK, Lassi ZS, Bhutta ZA. Adolescent health interventions: conclusions, evidence gaps, and research priorities. *J Adolesc Health* 2016; 59 (suppl 4): S88–92. 27664599 10.1016/j.jadohealth.2016.05.006 PMC5026678

[24] Weiss HA, Ferrand RA. Improving adolescent health: an evidence-based call to action. *Lancet* 2019; Published Online March 12. 10.1016/S0140-6736(18)32996-9. 30876704

[25] Lessler K. Health and education screening of school-age children–definition and objectives. *Am J Public Health* 1972; 62(2): 191–198. 10.2105/ajph.62.2.191 5058858 PMC1530305

[26] UNICEF. Child friendly school. 2009. Chapter 5: Schools as protective environments. Available at: http://www.unicef.org/education/files/CFSManual_Ch05_052009.pdf (accessed March 22, 2019).

[27] Singla D, Waqas A, Hamdani SU, Suleman N, Zafar SW, Zill-E-Huma, et al. Implementation and effectiveness of adolescent life skills programs in low- and middle-income countries: A critical review and meta-analysis. Behaviour Research and Therapy 2019.[Epub ahead of print]. 10.1016/j.brat.2019.04.010 31146889

[28] Winnard D, Denny S, Fleming T. Successful school health services for adolescents: best practice review. 2018.Available at: http://www.schoolnurse.org.nz/Attachments/pdf_files/bestpractice/Best_Practice_Best_Practice_SBHC_Review.pdf (accessed March 22, 2019).

[29] Norfazilah A, Hafizah Z, Siti Z, Azmawati MN. Poor peer support as a predictive factor towards depression among adolescent. Med & Health 2015; 10(1): 48–57.

[30] Jung HO, Kim HS. Effects of a positive peer relationship training program on self-esteem, bullying, and depression for children in early adolescence. *Child Health Nursing Research* 2014; 20(3):133–141.

