# Supplementary material for: School-based interventions to promote adolescent health: A systematic review in low- and middle-income countries of WHO Western Pacific Region
Source: PLoS One. 2020 Mar 5;15(3):e0230046. doi: 10.1371/journal.pone.0230046 (PMC7058297; doi:10.1371/journal.pone.0230046)
Supplement: S1 Table — (DOCX) [file pone.0230046.s001.docx]

Supporting information 1: Search strategy (Web of Science)

| Key words | Search strategy |
| --- | --- |
| School based | "School Health Services" OR "Students" OR "School Nursing" OR "Schools" OR pupil* OR school based OR school Children OR schoolchild* OR college* OR Classroom* |
| Adolescent | "Child" OR "Child Nutrition" OR "Child Health Services" OR "Child Nutritional Physiological Phenomena" OR "Child Mortality" OR "Child Welfare" OR "Child Care" OR "Child Reactive Disorders" OR "Child Guidance" OR "Child Reactive Disorders" OR "Child Behavior Disorders" OR "Child, Hospitalized" OR "Child Development" OR "Child Behavior" OR "Developmental Disabilities" OR "Mental Disorders Diagnosed in Childhood" OR "children" OR "young patient" OR "young patients" OR "Adolescent Behavior" OR "Adolescent Medicine" OR "Adolescent Psychiatry" OR "Psychology, Adolescent" OR "Adolescent, Hospitalized" OR "Adolescent, Institutionalized" OR "Adolescent Health Services" OR "Adolescent" OR "Young Adult" OR "Minors" OR Teenager* |
| WPRO LMICountries | "Cambodia" OR Cambodia* OR “Phnom Penh” OR Khmer OR Kamuchea* OR "China" OR China OR Chinese OR "Hong Kong" OR Macau OR Tibet* OR "Pacific Islands" OR "Melanesia" OR "Melanesia" OR "Polynesia" OR Fiji* OR “Suva” OR "Asia, Southeastern" OR Laos OR Lao OR Laotian OR Vientiane OR Hmong OR Borneo OR “Malaysia” OR Malaya* OR Malay OR Sabah OR Sarawak OR Penang OR “Kuala Lumpur” OR Kuching OR “Mekong Valley” OR Indochina OR “Philippines” OR “Vietnam” OR Vietnam* OR “Viet Nam” OR Hanoi OR “Ho Chi Minh” OR Manila OR Phillippines OR Philipines OR Phillipines OR Kiribati OR “Christmas Island” OR “Johnston Island” OR “Gilbert Islands” OR “Mariana Islands” OR Majuro OR Nauru OR “Pacific Islands” OR Tuvalu OR “Ellice Islands” OR “Caroline Islands” OR “Federated States of Micronesia” OR “Marshall Islands” OR Guam OR Agana OR Palau OR Koror OR “Mongolia” OR Mongolia* OR Ulaanbaatar. OR "Papua New Guinea" OR “Papua New Guinea” OR “East New Guinea” OR “New Britain” OR “New Ireland” OR “Admiralty Islands” OR “New Hanover” OR “ Bismarck Archipelago” OR Bougainville OR “Solomon Islands” OR “D'Entrecasteaux” OR “Trobriand Islands” OR “Woodlark Island” OR “Murua Island” OR “Louisiade Archipelago” OR “Solomon Islands” OR Papuan* OR Polynesian OR “New Caledonia” OR Vanuatu OR "Samoa" OR Samoa* OR “Navigator Island” OR “Navigator Islands” OR "Polynesia"[Mesh:NOEXP] OR "Pitcairn Island" OR "Tonga" OR Polynesia* OR “Pitcairn Island” OR Tonga* OR “Easter Island“ OR Niue OR Tokelau OR “Wake Island” OR “Wallis and Futuna” OR “Cook Islands” OR “Rarotonga” OR Tahiti |
| Intervention study | "Intervention" OR "program" OR Program* OR "trial" OR project |
