# Supplementary material for: School-based interventions to promote adolescent health: A systematic review in low- and middle-income countries of WHO Western Pacific Region
Source: PLoS One. 2020 Mar 5;15(3):e0230046. doi: 10.1371/journal.pone.0230046 (PMC7058297; doi:10.1371/journal.pone.0230046)
Supplement: S2 Table — (DOCX) [file pone.0230046.s002.docx]

###### Supporting information 2: Overview of study characteristics

| **Citation** | **Year of publication** | **Country** | **School setting** | **Participants** | **Research design** | **Health topics** | **Intervention Descriptions** | **Intervention characteristics** | **Outcome measures** | **Results and effect size** | **Lessons learnt** |
| --- | --- | --- | --- | --- | --- | --- | --- | --- | --- | --- | --- |
| Aplasca et al. | 1995 | Philippines | 4 public high schools (2 intervention and 2 control) in a semi-urban district | 804 high school students (420 intervention and 384 control)  Age: 13-16, average age is 14.7 in intervention and 14.9 in control. | CRCT | AIDS | Intervention Group: Teacher-led AIDS training program. Control Group: Reading materials.  Duration of intervention: 6 weeks. | 12 lessons, 40 mins twice a week for 6 week. The curriculum was designed to provide students with accurate information about AIDS, particularly in dispelling misconceptions about casual contagion, to foster positive attitudes towards people with AIDS and to develop skills aimed at clarifying values and assessing intended behaviour. | AIDS-related knowledge, attitudes; sexual behaviours and alcohol and drug use. | 1. The difference of changes in mean scores of AIDS knowledge is +0.45 (AIDS biology), +0.93 (transmission) and +0.41 (prevention), with P<0.01. 2. There was no statistically significant overall effect on intended preventive behaviour. | 1. Agreement and understanding by parents are essential; Pay attention to religious concerns. 2. AIDS prevention education curricula must aim to improve adolescents’ decision making skills. 3. Programs be taught and repeated regularly to ensure continuity and sustainability. 4. Integrate into regular school curricula and community programs |
| Xu et al. | 2000 | China | County level primary and secondary school | 4063 intervention and 1050 control.  Age: 6-12 for primary and 12-15 for secondary. | NRCT | Deworming-helminth infection/ HPS | Intervention Group: Examine and treatment of helminth infection, health education, improvement of physical environment, school policies and regulations.  Control Group: Examine and treatment of helminth infection.  Duration of intervention: 1 year. | Health education: added to teaching agenda and given once every 2 weeks; monthly wall newsletters; broadcasting in campus; brochures; video in class. Deworming: carried out twice during the intervention. Single dose of albendazole were used for most students; routine 3-day treatment of mebendazole was used for students with whipworm infection. Improvement of physical environment: improve the latrines and water supply facilities. School policies: develop policies for cleanliness of sanitary facilities, regular deworming, food safety and food hygiene. | 1. Students' knowledge and behaviours about helminth infection;  2. Prevalence of helminth infection in students; 3. Environmental egg contamination rate in school; 4. Change of physical environment (qualitative); 5. Change of school policies (qualitative); 6. Change of school psychosocial environment (qualitative). | 1. Knowledge passing rate increased from 10.9% to 82.7% (P<0.005) in the intervention group; percentage of 'good' health behaviour increased from 29.6% to 43.8% (P<0.01); no significant change in control group; 2. The multi-parasitism rate decreased from 42.8% to 7.3% (P<0.01) in the intervention group; no significant change in control group; 3. The egg contamination rate in the intervention group change from 22.4% to 4.3% (P<0.01), declining by 80.7%; however in the control group, the declining rate is only 27.7%. | 1. Targeting school for health intervention could result in large-scale health and education benefits. 2. The key elements for the intervention include active participation of all related sectors. 3. Helminth intervention is an excellent entry point for HPS programs in rural and semi-urban schools. 4. The concept of HPS is acceptable to government officials and community members. |

| **Citation** | **Year of publication** | **Country** | **School setting** | **Participants** | **Research design** | **Health topics** | **Intervention Descriptions** | **Intervention characteristics** | **Outcome measures** | **Results and effect size** | **Lessons learnt** |
| --- | --- | --- | --- | --- | --- | --- | --- | --- | --- | --- | --- |
| Xia et al. | 2004 | China | Urban and rural secondary school (3 interventions, 3 controls) | 4277 at baseline, 3346 at final evaluation.  Average age 13.7 in intervention group and 13.6 in control group. | NRCT | Nutrition/ HPS | Intervention Group: School-based working groups, nutrition training for school staff and students, student competitions, health promotion activities. Control Group: no specific activities.  Duration of intervention: 18 months. | School-based working group: Each intervention school established a working group, comprising of the headmaster, teachers, parents and community leaders. Nutrition training for students: health education class once every two weeks. Integrate instruction about nutrition-related topics into traditional academic subjects such as arts and language composition.  School policies: health-related school policies and the school environment, including renovations to school facilities and grounds. | 1. Nutrition knowledge; 2. Nutrition attitudes: 3. Nutrition behaviours | 1. The largest increases of knowledge occurred in the areas of nutrient-rich foods (increased from 36.0 to 59.6%) (P<0.01) and adequate dietary principles (increased from 61.9 to 85.8%) (P<0.01); 2. The attitude that 'nutrition is important for overall health' increased from 66.7% to 85.4% (P<0.01), 'the importance to eat three adequate meals each day' increased from 50.0% to 86.6% (P<0.01). 3. 'Washing hands before eating' increased from 66.4% to 89.8% (P<0.01), 'washing hands after using toilet' increased from 87.5% to 93.6% (P<0.01). | 1. School projects should seek close collaboration with institutions from the health and other sectors from the very beginning. 2. School policy and school environment is very important facilitator for program success. 3. Establishing nutrition and health education as a regular element of the school curriculum remains an important objective. 4. The HPS concept is feasible. It can effectively serve as an entry point to establish HPS in China. |
| Cartagena et al. | 2006 | Mongolia | Rural secondary school (8 interventions and 8 controls) | 320 intervention (M =43%, F=57%); 327 control (M =43.5%, F=56.5%).  Age 15-19. | NRCT | HIV & SRH | Intervention Group: Peer education on HIV and SRH. Control Group: no peer education.  Duration of intervention: 3 years. | 1. Peer educators (always both boys and girls) are chosen by local GTZ coordinators and teachers based on the following criteria; 2. The students who will become the peer educators are trained for a period of 3 days. The lesson plan is divided into 1) Reproductive health; 2) AIDS and STI transmission, symptoms and prevention; 3) Safe sex including how to use condoms; 4) Discussions and interactive communication through skills-based learning about emotions, refusal skills, love, and friendship. | 1. Knowledge (including identification of STI symptoms and methods of transmission and protection); 2. Attitude (towards HIV and SRH); 3. Self-efficacy (levels towards current or future sexual practices); 4. Behaviours (always condom use during the last three months of sexual intercourse). | 1. Intervention group had higher level of knowledge (RI 2.43; 95% CI 1.37-4.33), attitude (RI 1.78; 95% CI 1.22-2.61) and self-efficacy (RI 4.42; 95% CI 2.29-8.51), but no improvement in behaviours; 2. Educator teams of 1 or 2 peers for each class was more effective for knowledge (RI 5.03; 95% CI 3.08–8.21), attitude (RI 2.73; CI 95% 1.42–5.27), self-efficacy (RI 10-64, CI 8.59 –13.19) and practice (OR 3.80, CI 95% 2.26–6.41). | 1. Intervention effect on safe sex practices was not statistically significant, likely due to the small size of the sexually active subgroup; 2. Informal sexual health peer educational program was more effective than formal one; 3. Peer education program delivered by small teams were more effective in terms of behaviour change. |

| **Citation** | **Year of publication** | **Country** | **School setting** | **Participants** | **Research design** | **Health topics** | **Intervention Descriptions** | **Intervention characteristics** | **Outcome measures** | **Results and effect size** | **Lessons learnt** |
| --- | --- | --- | --- | --- | --- | --- | --- | --- | --- | --- | --- |
| Wen et al. | 2010 | China | Four junior high schools in Guangdong Province, 2 public and 2 private schools. | 2343 7th and 8th grade students, control (1004) and intervention (1339). Boys accounts for 52.1%, girls 45.9%.   Average age was 13.4. | NRCT | Tobacco use | Intervention Group: PRECEDE-PROCEED model intervention using socio-ecological framework.  Control Group: Basic once-a-year health curriculum.  Duration of intervention: 2 years. | Stage 1: Smoking prevention committee was established; non-smoking policy carried out; non-smoking signs and anti-smoking ads were posted on campus. Stage 2: 20-pages anti-smoking textbook edited; 8-h training workshop for school nurses and teachers; anti-smoking curriculum. Stage 3: Peer education using contest; parental letters for smoking-free family Stage 4: Peer education using contest; media advocacy in TV and newspaper. | 1. Students' knowledge & attitude' 2. Students' behaviour (have you ever tried cigarette smoking, even just 1 or 2 puffs? have you ever smoked cigarettes regularly, at least 1 cigarette every week for 3 months?) | The intervention increased knowledge in both 7th and 8th cohort, with the effect size being 0.32 in 7th cohort (P<0.001) and 0.41 in 8th cohort (P<0.001).  At the 1-year follow-up, the interventions reduced the probability of baseline experimental smokers’ escalating to regular smoker (7.9 vs 18.3%; adjusted odds ratio (OR) 0.34, 95% CI= 0.12–0.97), but did not reduce  the probability of baseline non-smokers’ initiating smoking (7.9 vs10.6%; adjusted OR 0.86, 95% CI =0.54–1.38). The intervention programme did not reduce the probability of smoking initiation (P>0.05). | 1. The first study to apply the socio-ecological framework and the PRECEDE– PROCEED model to prevent smoking among Chinese adolescents.  2. Diverse multi-level intervention components were combined to increase intervention effectiveness. 3. Some innovative yet feasible intervention components that deserved further testing and replication, including the demonstration of animal experiments, the signature of a public commitment not to smoke, smoke-free family contracts and communication with neighbouring stores that sold cigarettes. |
| Chen et al. | 2014 | China | Grade 1-2 middle school in Linzhi, Tibet Autonomous Region and Guangzhou, Guangdong Province | 709 Linzhi Tibetan (349 intervention, 360 control) and 1098 Guangzhou Han (592 intervention, 506 control).   Average age was 14.5±1.1 yeas, 50.4% were boys. | CRCT | Tobacco use | Intervention Group: Health policy in school; health environment in school and personal health skills.  Control Group: No specific intervention.  Duration of intervention: 1 year. | Health policies in school: tobacco control committees headed by the principal was established; regulations on smoking were made; Health environment in school: 30 no-smoking signs placed in the school yards. Peer education was conducted; group activities quarterly to discuss harms of smoking; Personal health Skills: Brochures, black board newsletter, class theme meetings, posters and publicity pictures, smoking-related health education lectures, smoking cessation related activities including essay competitions. | 1. Students' knowledge & attitude' 2. Students' behaviour (whether smoked, smoked daily, smoked weekly, or is smoking currently). | The intervention increased smoking-related knowledge in both Tibetan (β=1.32, 95% CI (0.87–1.77)) and Han ethnic groups (β =0.47, 95% CI (0.11–0.83)). Changed attitudes toward smoking in Tibetan (β =1.47, 95% CI (0.06–2.87)) but not so in Han (β=−0.33, 95% CI (−1.68–1.01)). No significant changes were detected in the prevalence of smoking. | 1. The currently adopted intervention was far from effective to decrease smoking behaviors.  2. The intervention intensity, for example, the frequency of health education lectures, Tobacco Control Group activities, could be increased.  3. Intervention can be more culture-related. Activities that embody collectivism could be designed.  4. More pertinent interventions targeting adolescents and sensitive to ethnic and cultural differences are greatly warranted. |

| **Citation** | **Year of publication** | **Country** | **School setting** | **Participants** | **Research design** | **Health topics** | **Intervention Descriptions** | **Intervention characteristics** | **Outcome measures** | **Results and effect size** | **Lessons learnt** |
| --- | --- | --- | --- | --- | --- | --- | --- | --- | --- | --- | --- |
| Jegannathan et al. | 2014 | Cambodia | 2 government secondary schools (1 intervention and 1 control), 6 classes in each school | 168 intervention (M = 92, F = 76); 131 control (M = 53, F = 78).   Data on age not available, Secondary school, young people. | NRCT | Suicide | Intervention Group: Life Skill modules related to suicide behaviours. Control Group: Three lessons on health, hygiene and nutrition (hand washing, micronutrient deficiency).  Duration of intervention: 6 months. | 1. The 6 Life skill modules include: Motivation, Concentration and improving memory; Problem solving skills; Peer pressure and saying ‘No’ to drugs/tobacco; Coping with stress, facing changes/problems; Self-esteem, sensation-seeking behaviour; Self-Awareness, understanding depression/suicide. 2. A core team comprised of teachers, psychologists, a psychiatric nurse and school nurses implemented the module. Weekly intervention sessions lasted 90–100 min, containing discussions, activities and home assignment. | 1. Attitude Toward Suicide (ATTS);  2. Youth Self Report (YSR); 3. Life Skills Development Scale-Adolescent Form (LSDS-AF). | 1. Effect size for YSR among all individuals showed no improvement. Among high-risk boys had a small to moderate effect size on Depressed (ES = 0.40), Attention problems (ES = 0.46), Aggressive behaviour (ES = 0.48) and Externalizing syndrome (ES = 0.64); 2. Girls showed improvement in Human Relationship (ES = 0.57), Health Maintenance  (ES = 0.20) and the Total Life Skills Dimensions (ES = 0.24), boys with high-risk behaviour improved on Human Relationship (ES = 0.48), Purpose in Life (ES = 0.26) and Total Life Skills Dimensions (ES = 0.22). | 1. Effect sizes indicate only mild to moderate improvement in Life Skills Dimension. Effect size for YSR-syndrome scores showed no improvement for either gender. 2. Gender specific intervention and focusing on high-risk individuals may lead to better results. 3. Teachers engaged in the program at the local level valued the process, indicating the possibility of scaling up the school-based mental health promotion at national level, provided the educational authorities are actively involved. |
| Mohammed Nawi A et al. | 2015 | Malaysia | Six secondary schools in Kuala Lumpur (4 intervention and 2 control) | 47 intervention (M=25, F=22); 50 control (M=30, F=20).  All participants were 12 years old. | CRCT | Obesity | Intervention Group: Website based obesity information, interactive toolbar in the website for discussion and comments. Respondents were also advised to measure BMI every 2 weeks.  Control Group: printed materials on the same information as in website.  Duration of intervention: 12 weeks. | 1.Participants and  parents in intervention group were provided with a website, which consists of information on healthy lifestyle, diet, and ways to overcome obesity. All respondents were signed up as a member in the website and were notified with any updates and information.  2. Interactive sessions were conducted in the chat toolbar in the website.  3. The respondents were advised to and calculate at home their BMI every two weeks or as required by using the BMI calculator provided in the website. | 1. BMI; 2. Waist circumference; 3. Body fat; 4. Quality of life, including physical, emotional, social and psychosocial functioning. | There was no significant reduction in BMI, waist circumference, and the body fat percentage between the intervention and control groups. The effect sizes of the reduction were too small (0.09, 0.11, and 0.09 for BMI, waist circumference and body fat percentage). | 1. The interactive web-based module had an impact on respondents who showed their commitment toward reducing their weight. Even though the intervention effect was small, it showed that technology played an important role in delivering the message compared to the routine pamphlets. 2.The consistency of health education and interactive sessions would be more beneficial and attractive to adolescents.  3.The study period of 12 weeks is considered short. A better impact can be discovered if a longer study period is used. |
